# Extractant Immobilization in Alginate Capsules (Matrix- and Mononuclear-Type): Application to Pb(II) Sorption from HCl Solutions

**DOI:** 10.3390/ma10060634

**Published:** 2017-06-09

**Authors:** Janette Alba, Ricardo Navarro, Imelda Saucedo, Thierry Vincent, Eric Guibal

**Affiliations:** 1 Departamento de Química, División de Ciencias Naturales y Exactas, Universidad de Guanajuato, Guanajuato, C.P. 36040, Mexico; janetac8@hotmail.com (J.A.); sauceti@ugto.mx (I.S.); 2 Centre des Matériaux des Mines d’Alès, Ecole des mines d’Alès, F-30319 Alès CEDEX, France; thierry.vincent@mines-ales.fr

**Keywords:** Cyanex 301, Cyanex 302, lead, uptake kinetics, sorption isotherms, desorption, matrix-encapsulation, mononuclear encapsulation

## Abstract

The decontamination of dilute industrial effluents is a critical challenge for decreasing the environmental impact of mining and metallurgical activities. As an alternative to conventional processes, new extractant impregnated resins (EIRs) have been synthesized by the immobilization of Cyanex 301 and Cyanex 302 in alginate capsules using two different procedures (matrix-type immobilization vs. mononuclear encapsulation). These materials have been tested for Pb(II) sorption from acidic solutions. The Langmuir equation fitted well the sorption isotherms and the maximum sorption capacities vary between 24 and 80 mg·g^−1^ at pH 1, depending on the type and loading of the extractant in the EIR. Uptake kinetics were controlled by the resistance to intraparticle diffusion; though both the Crank equation (intraparticle diffusion) and pseudo-second order rate equation equally fitted uptake profiles. The amount of extractant immobilized in mononuclear capsules is lower than in matrix-type beads; this leads to lower sorption capacities but slightly better mass transfer properties. The balance between the advantages and drawbacks of the different systems makes more promising matrix-type capsules. The desorption of Pb(II) is possible using 1 M HNO_3_ solutions: metal ions were completely desorbed. However, the probable oxidation of the extractants (conversion to oxidized forms more sensitive to pH) reduces the sorption efficiency when they are re-used.

## 1. Introduction

The international regulations concerning the discharge of metal ions to the environment and the incentive policies from national and intergovernmental institutions for recycling spent materials have driven great attention for the last decade on the development of new processes for metal recovery from low-concentration effluents.

A wide technical range exists for metal recovery: precipitation, solvent extraction, membrane processes, ion-exchange, and chelating resins. The selection of a suitable process is based on a series of criteria concerning the concentration range for metal, the flow rates, the value of the target metal, and the authorized discharged level. Some processes are facing technical limitations for reaching environmental regulations or produce huge amounts of wastes (metal-bearing sludge) that require specific and expensive handling and storage (controlled landfill): for example, precipitation methods have serious drawbacks due to target limits and to sludge production. Solvent extraction is very efficient for metal recovery [1,2,3,4]; however, the possible loss of extractants in the wastewater impacts both environmental benefits and economic competitiveness. This technique is generally reserved for the recovery of metal ions from relatively concentrated effluents (metal levels higher than 1 g·L^−1^). A new generation of extractants based on ionic liquids have also been designed that are quite promising [5]. Membrane processes generally perform well but require huge energy consumption [6,7]. Sorption processes involving ion-exchange or chelating resins [8,9,10,11,12,13,14,15], and biosorbents (mimicking synthetic resins by metal binding on the same kind of reactive groups) [16,17,18,19,20,21,22], or activated carbon [23,24] and inorganic materials [25,26,27] are alternative techniques usually more appropriate for low-concentration effluents.

An alternative process may consist in combining the high efficiency of solvent extraction with the stability benefits and easy operation of resin-based systems using impregnated materials [28]. Highly porous supports are impregnated with the extractant, which is immobilized in the opened porosity network [29,30,31,32,33,34,35,36,37,38,39]. Generally, the extractant is diluted in a solvent before impregnating the support, while in a second step the solvent is evaporated with simultaneous immobilization of the extractant. However, new processes have been recently developed for the immobilization of extractants and ionic liquids. The extractant is encapsulated in a polymer or a biopolymer matrix [40,41,42,43,44,45,46,47,48,49]. In this case, the extractant is generally immobilized in the matrix of the polymer by emulsification and subsequent polymerization or jellification (for example, ionotropic gelation of alginate in the presence of calcium ions), the so-called matrix-type encapsulation process (M). Recently, new processes have been developed for the immobilization of the extractant as a mononuclear liquid core coated by a thin layer of polymer; these materials are named mononuclear capsules (N).

The present study focuses on the recovery of lead from acidic solutions (such as the solutions produced from the acidic leaching of metal-containing wastes) using two thiophosphinic-based extractants (HA: Cyanex 301 and Cyanex 302) immobilized in alginate capsules prepared by the two processes (M and N) (Figure S1, See Supplementary Information). The extractant concentration in the extractant phase of the extractant impregnated resin (EIR) is varied. The effect of HCl concentration is investigated before determining the sorption isotherms and evaluating the contribution of diffusion mechanisms in the control of uptake kinetics. Finally, lead desorption from loaded sorbents is carried out. The main objective consists of selecting the best combination of extractant loading and the immobilization process.

## 2. Results and Discussion

### 2.1. Characterization of Sorbents

The optical microscopy observations showed some significant differences in the aspects of the beads that were roughly spherical for the two modes of synthesis. Indeed, for matrix-type capsules (M) the beads were opaque while mononuclear capsules (N) were transparent (Figure S2, See Supplementary Information). The mononuclear-type sorbents contain a liquid core of extractant (dissolved in kerosene at 50% or 75%) covered by a thin layer of biopolymer that makes light diffusion possible. On the other hand, the matrix-type sorbents are characterized by a gelled internal structure that does not allow light diffusion. The SEM-EDX (scanning electron microscopy coupled to energy dispersive x-ray spectroscopy) analysis of the capsules was performed on the sorbents. Figures S3 and S4 (See Supplementary Information) show examples of SEM images and the SEM-EDX analysis (for matrix-type material; the section of mononuclear-type beads releases the liquid core and the analysis of the cross-sections was not possible). For mononuclear-type beads, both the inner and outer surfaces are smooth and the thickness of the biopolymer layer ranges between 90 and 100 µm (for C301-N-50, Figure S3; See Supplementary Information). For matrix-type sorbents, the porous structure of the gelled internal part is clearly apparent; the element distribution map shows a homogeneous distribution of P element (tracer of the extractant) that is confirmed by its distribution profile along the cross-section: the intensity of the P signal remains roughly constant.

Table 1 reports the main characteristics of the sorbents: particle sizes range between 620 µm and 1850 µm. Cyanex content (*q*_HA_) in the beads increases with the Cyanex concentration in the organic phase. However, the Cyanex content also depends on the size of the beads: for C301-M-50 materials two different sizes of beads (L and S) were produced by changing the size of the nozzles in the Büchi Encapsulator; the amount of extractant is expected to be identical. However, large beads are characterized by a slightly higher content of HA (Cyanex extractant) than small beads. Nevertheless, large beads are less stable than small beads. Under selected conditions, the highest contents of the extractant in the beads were obtained with matrix-type encapsulation: large beads with 50% concentration (in kerosene) of Cyanex 301 reached 1.05 mmol C301 g^−1^ beads, while for Cyanex 302-sorbent extractant reached up to 1.57 mmol C302 g^−1^ beads (for 75% extractant concentration in the organic phase). 

The matrix-type mode of encapsulation allows for obtaining the highest loadings of the extractant in alginate capsules; for the same experimental conditions (type and size of nozzles, dilution of Cyanex, etc.). However, beads with high extractant content are less stable. Cyanex 301 and Cyanex 302 reached very similar extractant loadings (1.05 and 1.03 mmol Cyanex g^−1^ beads, respectively) for matrix-type capsules with 50% extractant concentration in the organic phase.

### 2.2. Influence of HCl Concentration on Pb(II) Sorption

**Figure 1:**
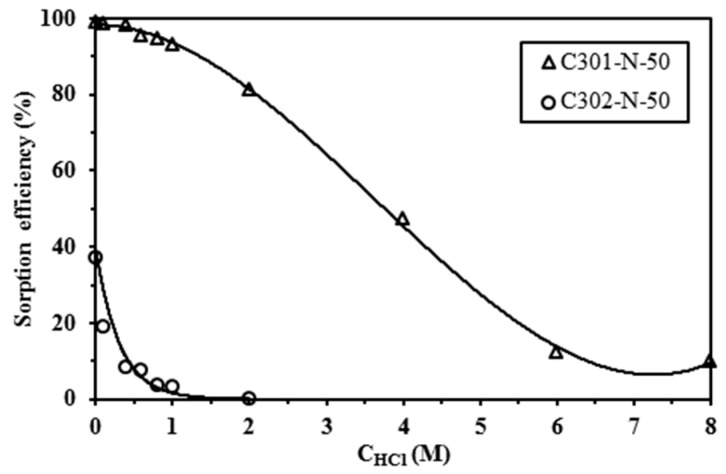
Effect of HCl concentration on the sorption of Pb(II) using Cyanex 301 and Cyanex 302 encapsulated in alginate mononuclear capsules (sorbent dosage, *SD*: 2 g·L^−1^; contact time: 4 days; *T*: 20 °C; *C*_0_: 50 mg·Pb·L^−1^; extractant concentration in kerosene: 50% v/v; agitation speed, v: 150 rpm).

The influence of HCl concentration on Pb(II) sorption was tested on two sorbents (mononuclear alginate capsules containing Cyanex 301 and Cyanex 302). The acid concentration was varied between 0.01 M and 8 M (Figure 1). First, the figure shows that under similar experimental conditions the Cyanex 301-based material was significantly more efficient than the Cyanex 302-sorbent: in 0.01 M HCl solutions Cyanex 301-sorbent achieved the complete recovery of Pb(II) while the sorption efficiency did not reach 40% for Cyanex 302-sorbent. In addition, Cyanex 301-sorbent is much less sensitive to the HCl concentration. Indeed, in 1 M HCl the sorption efficiency drastically decreased to 3% for the Cyanex 302-sorbent as opposed to the Cyanex 301-sorbent that maintained its sorption efficiency close to 93%. However, when the HCl concentration exceeds 2 M the sorption efficiency of Cyanex 301-sorbent drastically decreased to 50% in 4 M HCl solutions and down to 15% in 6 M HCl solutions. Similar trends were reported for Cd(II) extraction using Cyanex 301 and Cyanex 302 [50]. Sole and Hiskey [51] reported the general reaction involved in the solvent extraction of M*^n+^* with mono- and dithioorganophosphinic acids (HA, such as Cyanex 301 and 302):

(1) $$M^{n+}\;+\;\frac{m+n}{p}\;{\left(\mathrm{HA}\right)}_{p,org}↔\;{\left({\mathrm{MA}}_{n}{\left(\mathrm{HA}\right)}_{m}\right)}_{org}\;+\;n{\;H}^{+},$$

Increasing the concentration of acid obviously tends to displace the equilibrium toward the dissociation of the complex between the metal cation and the organophosphinic acid. The stronger effect of HCl concentration on Cyanex 302-sorbent is probably explained by the highest pk_a_ of the extractant (i.e., 5.63 vs. 2.61 for Cyanex 301, [51]). In addition, Staszak et al. [50] discussed the FT-IR changes of the organic phase after Cd(II) extraction and they suggest that the HCl molecules are transported to the organic phase by a solvating mechanism or by the direct coordination with free ligands; in any case, these possible mechanisms contribute to blocking the complexing centers and to reducing the affinity of the extractant for metal ions.

Further studies were performed using 0.1 M HCl solutions in order to maintain a high relative sorption of Pb(II) with both the Cyanex 301-sorbents and Cyanex 302-sorbents.

### 2.3. Sorption Isotherms

Sorption isotherms were carried out at pH 1 (0.1 M HCl solutions) in order to: (a) evaluate the affinity (coefficient *b* of the Langmuir equation) and the maximum sorption capacity (*q*_m_) of the different sorbents; (b) compare the efficiencies of the Cyanex 301 and Cyanex 302 extractants for both matrix-type (M) and mononuclear-type (N) encapsulation modes. Varying the amount of extractant immobilized in the encapsulated materials allowed for evaluating the apparent stoichiometry between the extractant and metal ions at saturation of the sorbents.

**Figure 2:**
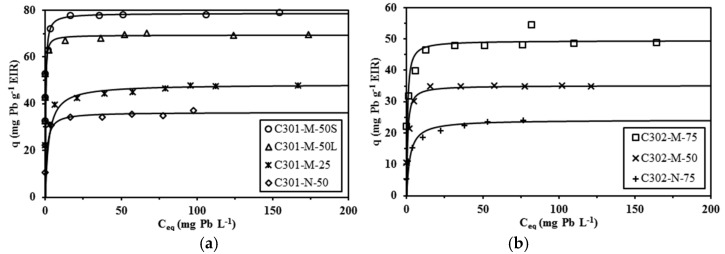
Pb(II) sorption isotherms using Cyanex 301 (**a**) and Cyanex 302 (**b**) encapsulated in alginate capsules (M: matrix-type capsules; N: mononuclear capsules) (solid lines: modeling of sorption isotherms with the Langmuir equation and coefficients reported in Table 2) (*C*_HCl_: 0.1 M; contact time: 4 days; *T*: 20 °C; *v*: 150 rpm).

Figure 2 shows the Pb(II) sorption isotherms for Cyanex 301-sorbents and Cyanex 302-sorbents. All the curves were characterized by a steep initial slope and a saturation plateau reached to a residual Pb(II) concentration close to 50 mg·Pb·L^−1^. This asymptotic trend is characteristic of the Langmuir-type equation, contrary to the power-type trend followed by the conventional Freundlich equation. Therefore, the sorption isotherms were only modelled using the Langmuir equation and the parameters of the model, for the different systems, are reported in Table 2. In Figure 2 the solid lines represent the modeling of sorption isotherms with the Langmuir equation and the parameters reported in Table 2: the experimental data are well fitted by the Langmuir equation. It is noteworthy that the Langmuir equation is supposed to describe the sorption of the solute as a monolayer on sorption sites of identical energy (and without interactions). The fit of experimental data does not necessarily mean that the hypotheses of the model are strictly verified. In the present case, the encapsulating material (alginate) has a specific affinity for metal cations; however, at the selected pH (close to 1) the sorption affinity of alginate for Pb(II) strongly decreases with increasing acidity (due to protonation of reactive groups; i.e., carboxylic groups) [52,53]. This is consistent with Pb(II) binding mainly processing through the interaction with the Cyanex extractant; this is also consistent with the homogeneity of the materials at least in terms of the reactive sites (fixed and constant energy for reactive groups).

Mononuclear-type beads have significantly lower maximum sorption capacities than matrix-type beads: for the same dilution ratio of the extractant (in the mixture extractant/diluent) the maximum sorption capacity was halved for mononuclear-type beads. As expected, increasing the extractant concentration in the organic phase increased the effective extractant content in the beads and as a consequence the sorption capacity of the sorbent was improved. The variations of the affinity coefficient (i.e., coefficient *b*) are more difficult to correlate to the characteristics of the sorbents.

The molar ratio Cyanex/Pb(II) at saturation of the sorbents (i.e., molar ratio *q*_HA_/*q*_m_) was relatively homogeneous for the Cyanex 302-based sorbents: it ranges between 5.69 and 6.57 mol/mol (mean value: 6.11). In the case of Cyanex 301-based materials, the variations are much larger (1.66–3.14) with a mean value equal to 2.27. Investigating the extraction of Pb(II) with Cyanex 301 (diluted in kerosene with 10% v/v n-decanol) Facon et al. [1] reported that PbL_2_ (i.e., Pb(Cyanex 301)_2_) is the complex extracted in the organic phase. Similar conclusions were reached by Argekar and Shetty for Pb(II) extraction with Cyanex 302 [54]. These results are consistent with those obtained with Cyanex 301 based beads. However, in the case of Cyanex 302 based beads, the molar ratio Cyanex/Pb(II) is much higher (6.11). This suggests that different species are extracted (i.e., PbA_2_(HA)_4_) or that the extractant is not efficiently (or completely) used. Moreover, increasing the amount of extractant tended to decrease the efficient use of the extractant. For the same extractant concentration in kerosene, mononuclear-type beads (lower effective Cyanex content) present a more efficient use of the extractant. The mononuclear encapsulation improves the rational use of the extractant.

The sorption capacities obtained with these materials are lower compared to the values reported in the literature that may reach 150–250 mg·Pb·g^−1^; however, this comparison is not sensible since the great majority of published papers focus on the sorption of Pb(II) from solutions in which the pH was controlled to a pH 4–6 range. It is noteworthy to reiterate that the objective of the study consists of recovering lead from acid solutions (such as those that are produced during the acid leaching of metal-containing wastes).

### 2.4. Uptake Kinetics

Uptake kinetic profiles have been obtained in 0.1 M HCl solutions (sorbent dosage, SD: 2 g·L^−1^; initial concentration: 170 mg·Pb·L^−1^). The analysis of the mechanisms controlling the sorption kinetics was performed using the specific plots corresponding to the homogeneous diffusion model (HDM) and the shrinking core model (SCM) associated to resistance to film diffusion, resistance to intraparticle diffusion, and control by the chemical reaction rate [55]. The detailed analysis of experimental data is presented in the Supplementary Information (Figures S5–S11). Though the testing of the models does not show a clear and uniform trend for the different systems, in most cases the resistance to intraparticle diffusion appears to be the most significant controlling mechanism (regardless of the general model; i.e., HDM or SCM). The structure of the beads is expected to affect the diffusion regime: matrix-type beads (M) are relatively homogeneous in structure as shown in Figure S4 (See Supplementary Information) while mononuclear-type beads (N) are considered as heterogeneous systems (a 100 µm-thin layer coating the liquid phase). This physical difference is not clearly confirmed by the comparison of the models for M-type and N-type.

**Figure 3:**
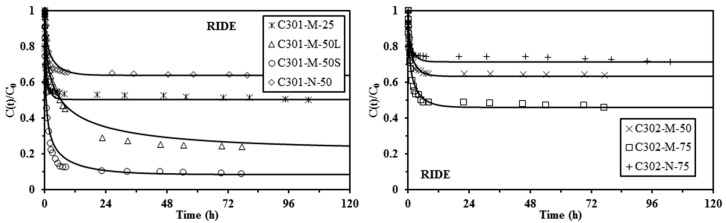
Pb(II) uptake kinetics using Cyanex 301 and Cyanex 302 immobilized in alginate capsules (M: matrix-type capsule; N: mononuclear capsule) (solid lines: modeling of uptake kinetics with the RIDE—Crank equation—and coefficients reported in Table 3) (*C*_0_: 170 mg·Pb·L^−1^; *SD*: 2 g·L^−1^; *C*_HCl_: 0.1 M; *v*: 150 rpm; *T*: 20 °C).

**Figure 4:**
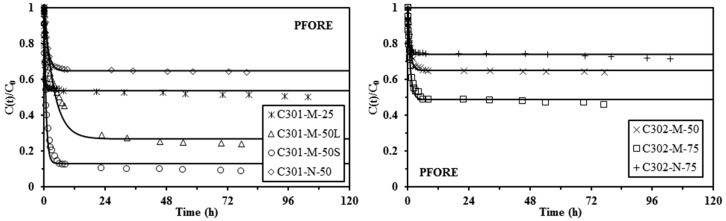
Pb(II) uptake kinetics using Cyanex 301 and Cyanex 302 immobilized in alginate capsules (M: matrix-type capsule; N: mononuclear capsule) (solid lines: modeling of uptake kinetics with the PFORE - and coefficients reported in Table 4) (*C*_0_: 170 mg·Pb·L^−1^; *SD*: 2 g·L^−1^; *C*_HCl_: 0.1 M; *v*: 150 rpm; *T*: 20 °C).

**Figure 5:**
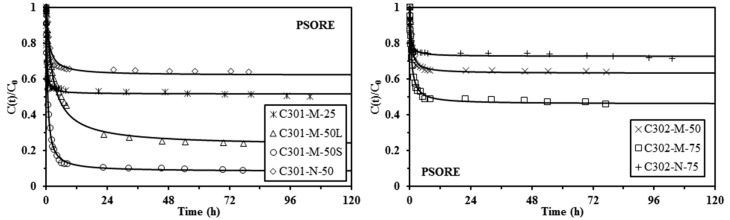
Pb(II) uptake kinetics using Cyanex 301 and Cyanex 302 immobilized in alginate capsules (M: matrix-type capsule; N: mononuclear capsule) (solid lines: modeling of uptake kinetics with the PSORE - and coefficients reported in Table 4) (*C*_0_: 170 mg·Pb·L^−1^; *SD*: 2 g·L^−1^; *C*_HCl_: 0.1 M; *v*: 150 rpm; *T*: 20 °C).

Figure 3, Figure 4 and Figure 5 show the uptake kinetics for the different sorbents: the solid lines represent the fit of the experimental curves with the resistance to intraparticle diffusion (RIDE), the pseudo-first order rate equation (PFORE), and the pseudo-second order rate equation (PSORE), respectively. The corresponding parameters for the models are summarized in Table 3 and Table 4. 

Figure 3 confirms that the RIDE models roughly fit the experimental data: the model was generally superimposed to experimental points; however, in some cases (for example, for the Cyanex 301-based sorbents) the RIDE fails to fit the curvature of the kinetic profiles. This is probably due to the simultaneous contribution of other resistance mechanisms (for example, resistance to film diffusion). The intraparticle diffusion coefficient varies in the range from 0.27 × 10^−11^–9.26 × 10^−11^ m^2^·min^−1^ for the Cyanex 301-based sorbents and in the range from 2.40 × 10^−11^–7.13 × 10^−11^ m^2^·min^−1^ for the Cyanex 302-based sorbents. These values are about three orders of magnitude lower than the value reported for the molecular diffusivity of Pb(II) in water (i.e., 4.8 × 10^−8^ m^2^ min^−1^, [56]). The comparison of kinetic profiles for small and large Cyanex 301-M-50 sorbents clearly shows that the equilibrium is reached significantly faster for small particles, as more evidence of the impact of resistance to intraparticle diffusion on the control of uptake kinetics.

Table 4 reports the parameters of the PFORE and PSORE models. The quality of the fit of the kinetic profiles can be “measured” through two criteria: (a) the value of the mean square of residuals (MSR); (b) the comparison of the experimental and calculated values of equilibrium sorption capacity. Except for the C301-N-50 sorbent, the MSR was systematically lower for the PSORE than for the PFORE and *q*_e,calc_ was closer to *q*_e,exp_ for PSORE than for PFORE. The PSORE is thus generally more appropriate for describing kinetic profiles for Pb(II) sorption using C301-based sorbents and C302-based sorbents. When increasing the extractant concentration in the organic phase, the effective Cyanex content in the sorbent is increased and the apparent rate coefficient for PSORE tends to decrease for both Cyanex 301 and Cyanex 302 based sorbents (Matrix-type). In addition, for the same fraction of extractant in the organic phase, the apparent rate constant for PSORE is greater for mononuclear-type sorbents (lower effective Cyanex content). It is noteworthy that this trend should be taken as indicative; indeed, the extractant content in the beads changes the saturation reached under selected experimental conditions and, consequently, should affect the mass transfer properties.

The amount of Cyanex immobilized (*q*_HA_) in mononuclear-type capsules is lower than in matrix-type capsules. As a consequence, the residual Pb(II) concentrations at equilibrium are much higher than those of the matrix-type capsules and the required time for reaching equilibrium is reduced.

### 2.5. Metal Desorption and Sorbent Recycling

Previous studies have shown that C301-M-25, C302-M-50, and C302-N-75 systems have greater stabilities and the fastest uptake kinetics among the different systems. Although the sorption capacities were not necessarily the highest, these systems were preferred for shortening the contact times and for improving the stability of the EIRs along the different sorption/desorption cycles. 

**Figure 6:**
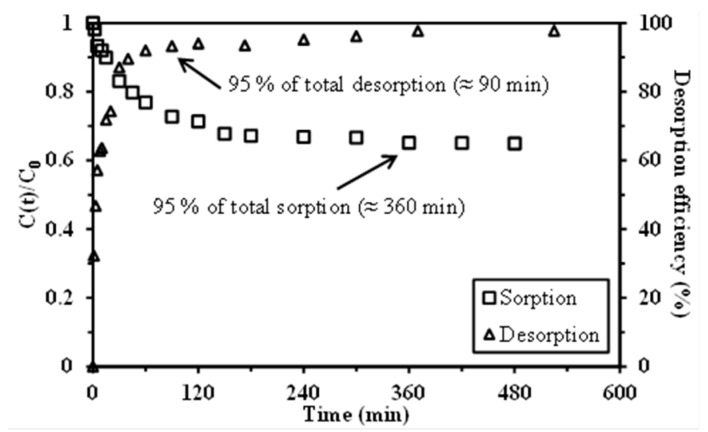
Comparison of Pb(II) sorption and desorption kinetic profiles for C302-M-50 sorbent.

The desorption of Pb(II) from metal-loaded sorbents was initially tested using nitric acid and thiourea acidic solutions for selecting the most appropriate eluent. Nitric acid solutions were tested at 0.1 M and 1 M concentrations. Argekar and Shetty [54] used Cyanex 302 for the solvent extraction of Pb(II) and 0.1 M HNO_3_ solutions for metal stripping from loaded organic phases. Thiourea in HCl solutions have also been frequently used for the stripping of metal ions (especially noble metals [57,58,59]) from loaded organic phases for the desorption of metal ions from impregnated resins and sorbents: 1 M thiourea in 1 M HCl solution was also carried out. Table 5 reports the results for Pb(II) desorption from selected metal-loaded sorbents (i.e., C301-M-25, C302-M-50, and C302-N-75). These tests clearly show that, regardless of the sorbent, HNO_3_ solutions have strong potential for desorbing Pb(II); however, a 1 M concentration is needed for achieving complete Pb(II) desorption. With an acidic thiourea solution, the desorption efficiency ranged between 83% and 88%. In addition, the recovery of Pb(II) from thiourea solutions is more difficult (for example by electrodeposition) than from simple acidic solutions. These two reasons make it preferable to use 1 M nitric acid solutions for Pb(II) recovery from loaded sorbents. The kinetics of desorption was performed on C302-M-50 capsules and compared to the kinetic profile for the sorption step (Figure 6). Under selected experimental conditions, the maximum desorption efficiency reached 97.7% after 9 h of contact. This test confirms the highly efficiency of nitric acid (1 M) for desorption of Pb(II). However, 95% of total desorption occurred within the first 90 min of contact (to be compared to 360 min of contact for reaching 95% of total sorption). Thirty-five min of contact were necessary for reaching 90% of total desorption. The kinetics of desorption is slightly faster than the kinetics of sorption.

The next step of the study investigated the recycling of the sorbent along three cycles of sorption and desorption using 1 M HNO_3_ solutions (and a rinsing step between every operating step using 0.01 M HCl solution). Table 6 reports for C301-M-25, C302-M-50, and C302-N-75 capsules the sorption and desorption efficiencies for the three cycles. All the Cyanex/alginate capsules follow the same trend: (a) the first sorption/desorption cycle operates well with complete desorption of the loaded Pb(II); and (b) the second step shows a very depreciated sorption of Pb(II); the desorption is almost complete but the low metal loading makes the discussion less meaningful. This may be explained by the progressive oxidation of the extractants in the presence of oxidant solutions of nitric acid. Indeed, oxidant acid solutions contribute to the oxidation of Cyanex 301, which is progressively converted to Cyanex 302, which, in turn, may be transformed into Cyanex 272 [60]. Cyanex 272 has a good affinity for extracting metal cations; however, the optimum pH range is shifted toward higher pH values, due to a higher pk_a_ value (6.37 against 5.63 and 2.61 for Cyanex 302 and Cyanex 301, respectively) [51].

## 3. Materials and Methods 

### 3.1. Materials

Cyanex 301 (bis(2,4,4-trimethylpentyl)dithiophosphinic acid extractant) and Cyanex 302 (bis(2,4,4-trimethylpentyl)monothiophosphinic acid extractant) were purchased from Cytec (Woodland Park, NJ, USA) and used as supplied without purification; the purity of the extractants is reported to be in the range 80–85%. Figure S1 shows the chemical structure of the extractants (See Supplementary Information). Kerosene (Fluka AG, Buchs, Switzerland) was used for diluting the extractants. Alginate was supplied by FMC Biopolymers (Philadelphia, PA, USA): the characteristics of the biopolymer have been previously determined [61]: the fractions of guluronic and mannuronic residues were: 67/33, respectively. Other reagents (CaCl_2_, PbCl_2_ etc.) were analytical grade and supplied by Sigma-Aldrich (Saint-Quentin Fallavier, France).

### 3.2. Encapsulation Procedures

Two procedures were used for the synthesis of EIRs: 

The matrix-type EIRs were obtained by the preparation of a stable emulsion associating the aqueous alginate solution and the extractant (diluted in kerosene, changing the extractant/kerosene fractions) and distributing the emulsion in a 0.5 M CaCl_2_ solution through a nozzle using the Encapsulator E-390 (Büchi, Switzerland).

The mononuclear-type EIRs were prepared with the encapsulator using the concentric nozzles system (the alginate solution is pumped through the shell circuit, while the extractant solution is pumped through the core circuit). The beads were ionotropically gelled in a 0.5 M CaCl_2_ solution.

The encapsulator uses a combination of “tools” for preventing the aggregation of the beads (electrostatic potential and vibration of the nozzles system) and for enhancing the homogeneity of bead size (size of nozzles, controlled pressure of extrusion, flow rates of emulsion, and flow rates of alginate (shell) and extractant (core) circuits, etc.). The optimization of the extrusion process requires pre-adjustment of the settings of these different tools. The values selected for the different parameters (alginate concentration, flow rates, pressure, vibration frequency, electrostatic potential, nozzle diameters, etc.) are reported in Table S1 (See Supplementary Information). For example, the acidity and the viscosity of Cyanex 301 and Cyanex 302 extractants are substantially different: (a) Cyanex 302 is more viscous than Cyanex 301 (19.5 vs. 7.8 kg·m^−1^·s^−1^, respectively); and (b) Cyanex 301 has a lower pk_a_ than Cyanex 302 (2.61 vs. 5.63, respectively) [51]. 

The stable emulsion was prepared by mixing 130 mL of alginate solution (1.5%, w/w) with 70 mL of extractant during 10 min (using an UltraTurrax at the speed of 11,000 rpm). The proportion of each extractant was modified to prepare microcapsules with different loadings (25–75% v/v in kerosene). The microcapsules were maintained in the coagulation solution one day before being rinsed with 0.1 M HCl solution. They were stored in 0.1 M HCl, in order to prevent possible degradation of the microcapsules.

### 3.3. Characterization of Encapsulated Materials

Optical photographs were obtained using a Leica Wild M10 microscope (Leica, Wetzlar, Germany). The morphology and the distribution of elements in the materials were determined with a Scanning Electron Microscope coupled with Energy Dispersive X-ray analysis (SEM-EDX). SEM observations were performed using an Environmental Scanning Electron Microscope (ESEM) Quanta FEG 200 (FEI, Thermo Fisher Instruments, FEI France, Merignac, France), equipped with an OXFORD Inca 350 Energy Dispersive X-ray (EDX) microanalysis system (Oxford Instruments, Abingdon, United Kingdom). The use of environmental SEM allowed for the direct observations of materials, without previous metallization of the samples. The topography of the samples was observed using secondary electron flux while the backscattered electrons were used for the identification and localization of heavy metals at the surface of the materials (by phase contrast). SEM-EDX facilities were used for the detection of elements and their semi-quantitative analysis: for example using P element as the tracer of the extractants. The standard accelerating voltage was set at 12.5 kV. The samples were analyzed on freshly cut sections.

SEM and SEM-EDX characterization was performed on freeze-dried materials. The freeze-drying has a strong degrading impact on the structure of the beads: the extractants tend to flow out the beads that lose their structure. As a consequence SEM and SEM-EDX analyses were only performed on metal-loaded matrix-type EIR (a cross-section of mononuclear EIR is also presented). 

The concentration of the extractant in the beads was determined by mineralization of the EIRs and P element analysis. A fixed amount of EIR (close to 30 mg, wet weight) was mixed with 2 mL of sulfuric acid under heating (with reflux) for 20 min. After decreasing the temperature, 1 mL of hydrogen peroxide (30% w/w) was added under heating for another 15 min. When necessary, 1 mL of hydrogen peroxide was added again, until complete discoloration of the solution (which passed from yellowish to uncolored). Finally, the volume of digested material was adjusted to 25 mL with Milli-Q water and the final solution was analyzed by ICP-AES (inductively coupled plasma atomic emission spectrometry, ICP Jobin-Yvon Horiba JY Activa-M, Longjumeau, France) for P content (P being the tracer of the extractants).

### 3.4. Sorption Procedures

Sorbent particles were used as wet material. A fixed amount of EIR (m, g) was mixed with a known volume (V, L) of Pb(II) solution (initial concentration *C*_0_, mg·Pb·L^−1^) prepared in HCl solutions (fixed concentration) under agitation (150 rpm). Relevant experimental conditions are systematically reported in the caption of the figures. After filtration (membrane filtration unit: 1 µm pore size) the samples were analyzed for residual metal concentration (*C*_eq_, mg·Pb·L^−1^) by ICP-AES, and the mass balance equation was used for calculating the concentration of the metal in the sorbent (*q*, mg·Pb·g^−1^): *q* = (*C*_0_ − *C*_eq_)*V*/*m*. Sorbent dosage (*SD* = *m*/*V*), extractant content, and metal concentration in the sorbent are reported considering the wet basis.

Sorption isotherms were obtained in 0.1 M HCl by contact of the sorbent with lead solutions varying the initial concentration between 0 and 300 mg·Pb·L^−1^ (sorbent dosage: 2 g·L^−1^, except for C302-N-75 with *SD*: 4 g·L^−1^), and the contact time was set to 4 days with a temperature close to 20 °C. Uptake kinetics were obtained using 170 mg·Pb·L^−1^ solutions (in 0.1 M HCl solutions) with a sorbent dosage of 2 g·EIR·L^−1^ and withdrawing samples at fixed contact times. Detailed experimental conditions are systematically reported in the caption of the figures.

Sorption processes are more adequate for the treatment of dilute solutions compared to solvent extraction and precipitation that are more appropriate for the recovery or removal of metal ions from concentrated effluents. When the solution is too concentrated the concentrating factor expected from the steps of sorption and desorption is too low to make the process competitive. The sorption process is generally meaningful for the treatment of concentrations below 200 mg·L^−1^. In the present study, the concentrations were selected close to 50 mg·L^−1^ for the study of pH effect and 170 mg·L^−1^ for the study of uptake kinetics, although higher concentrations were used for establishing the sorption isotherms and determining the maximum sorption capacities.

Desorption was performed by mixing an amount of 20 mg of EIR with 10 mL of Pb(II) solution (0.1 M HCl, initial metal concentration: 130 or 200 mg Pb(II) L^−1^) for 24 h. The residual concentration, measured by ICP-AES after resin separation, served to determine the amount of metal bound to the resin. The metal loaded resin was mixed for 24 h with 10 mL of different eluents: 0.1 M HNO_3_ solution, 1 M HNO_3_ solution, and 1 M thiourea in 1 M HCl solution. After resin separation, the metal concentration in the eluent was determined by ICP-AES, and the amount of lead desorbed was used for calculating the Pb desorption efficiency. For the study of resin recycling, a rinsing step was carried out, using a 0.01 M HCl solution, before using the resin for the next sorption step. For the evaluation of sorption/desorption cycles, the same procedure was used for three cycles. Desorption kinetics were also investigated. The sorbent was loaded with Pb(II) by contact of 1 g of sorbent with 500 mL of 200 mg Pb(II) L^−1^ solutions (in 0.1 M HCl solution) at room temperature under 150 rpm agitation for 24 h. After solid/liquid separation the Pb(II)-loaded capsules were maintained for 24 h, at room temperature, under agitation (at 150 rpm) with 500 mL of 1 M HNO_3_ solutions. Samples were collected at different contact times and the Pb(II) concentration was determined by ICP-AES for calculating the desorption efficiency: *DE*(%) = 100(1 − *q*(t)/*q*_0_).

### 3.5. Sorption Modeling

Sorption processes are controlled by a series of mechanisms such as the proper reaction rate and resistance to bulk diffusion, to film diffusion, and to intraparticle diffusion [62]. In most cases, appropriately mixing the suspension makes the resistance to bulk diffusion negligible and contributes to reducing the impact of resistance to film diffusion. Previous studies on extractant impregnated resins [30], and composite materials associating alginate and extractants [63], or ionic liquids [64] have confirmed the predominating impact of resistance to intraparticle diffusion on the control of uptake kinetics. A more complete discussion of the mechanisms that control uptake kinetics is presented in the Supplementary Information with the integration of a homogeneous diffusion model and shrinking core model with resistance to film diffusion, resistance to intraparticle diffusion, and with control by the chemical reaction rate [55,65,66].

The intraparticle diffusion coefficient (*D*_e_, effective diffusivity, m^2^·min^−1^) was determined using the Crank equation (Equation (2)), assuming the solid to be initially free of metal, and the kinetics to be controlled by resistance to intraparticle diffusion [67]:

(2a) $$\frac{q\left(t\right)}{q_{\mathrm{eq}}}=1-\overset{\infty }{\underset{n=1}{\sum }}\frac{6\alpha \left(\alpha +1\right)\exp\left(\frac{-D_{e}q_{n}^{2}t\;\;}{r^{2}}\right)}{9+9\alpha +q_{n}^{2}\alpha^{2}},$$

*q*(t) and *q*_eq_ are the concentrations of the metal in the resin at time t and equilibrium, respectively, *r* is the radius of the particle, and *q*_n_ non-zero roots of the equation:

(2b) $$\tan q_{n}=\frac{3q_{n}}{3+\alpha q_{n}^{2}},$$

with

(2c) $$\frac{mq}{VC_{0}}=\frac{1}{1+\alpha },$$

The Mathematica™ software (version.4, Wolfram, Wolfram France, Paris, France) was used for the determination of the intraparticle diffusion coefficient, *D*_e_, and for the simulation of the experimental data (represented by the continuous line on the figures).

In addition, the pseudo-first order rate equation (PFORE; i.e., the Lagergren equation) and the pseudo-second order rate equation (PSORE) have been tested for the modeling of the experimental uptake kinetics [68]. These models have been initially designed for the modeling of reaction kinetics in homogeneous systems. However, these models are also frequently used for describing kinetic profiles in solid/liquid separation. As a consequence, the kinetic parameters should be considered as an apparent rate constant: indeed, the contribution of diffusion mechanisms is fully integrated in the apparent rate constants.

PFORE:

(3a) $$\frac{dq\left(t\right)}{\mathrm{dt}}\;=\;k_{1}\left(q_{\mathrm{eq}}-q\left(t\right)\right),$$

after integration (with appropriate boundary condition: *q*(0) = 0)

(3b) $$\ln\left[1-\frac{q\left(t\right)}{q_{\mathrm{eq}}}\right]=-k_{1}t,$$

or

(3c) $$q\left(t\right)=\;q_{\mathrm{eq}\;}\left[1-\mathrm{Exp}\left[-k_{1}t\right]\right],$$

*k*_1_ (min^−1^) is the apparent rate constant for the PFORE.

PSORE:

(4a) $$\frac{dq\left(t\right)}{\mathrm{dt}}\;=\;k_{2}{\left(q_{\mathrm{eq}}-q\left(t\right)\right)}^{2},$$

after integration (with appropriate boundary condition: q(0) = 0)

(4b) $$\frac{t}{q\left(t\right)}=\;\frac{1}{k_{2}q_{\mathrm{eq}}^{2}}\;+\;\frac{1}{q_{\mathrm{eq}}}t,$$

*k*_2_ (g·mg^−1^·min^−1^) is the apparent rate constant for the PSORE.

In order to avoid possible statistical bias (which may occur when using linear regression with linearized equations) the parameters of the models (i.e., *q*_eq,calc_ and *k*_1_ for PFORE, *q*_eq,calc_ and *k*_2_ for PSORE) were obtained by non-linear regression using the Mathematica^®^ software package.

Equilibrium distribution of the solute between liquid and solid phases is usually described by the sorption isotherm (plot of *q*_eq_ vs. *C*_eq_). Many equations have been developed for modeling sorption isotherms: the most commons are the Langmuir and the Freundlich equations [62]. While the Langmuir equation is a mechanistic equation, the Freundlich equation is an empirical power-type function. The Langmuir equation assumes the sorption to occur as a monolayer at the surface of the sorbent through homogeneous interactions with the solute (homogeneous energy of sorption between reactive groups and solute molecule), and without interactions between sorbed molecules. Based on the shape of the sorption isotherms (see below), which are characterized by an asymptotic saturation trend, the equilibrium will be preferentially described by the Langmuir equation (Equation (5)):

(5) $$q_{\mathrm{eq}}=\frac{q_{m}b\;C_{\mathrm{eq}}}{1+bC_{\mathrm{eq}}},$$

where *q*_m_ (mg·Pb·g^−1^) is the sorption capacity at saturation of the monolayer (to be compared with the experimental maximum sorption capacity) and *b* (L·mg^−1^) is the affinity coefficient (i.e., ratio between the sorption and desorption rate coefficients).

## 4. Conclusions

The immobilization of Cyanex 301 and 302 extractants in alginate capsule can be efficiently operated by using two techniques: (a) the matrix-mode (which consists of the ionotropic gelation of the emulsion between the aqueous alginate solution and the organic phase constituted by the extractant and kerosene); and (b) the mononuclear-mode (which consists of the coating of a drop of extractant dissolved in kerosene by a 100 µ-thin layer of alginate gel, as shown by the SEM analysis). SEM and SEM-EDX analyses showed an internal porosity and a homogeneous distribution of the extractant in the matrix-type capsules. 

The extractants are efficient for recovering Pb(II) in weakly acid solutions (i.e., 0.1 M HCl): Cyanex 301 is less sensitive to pH than Cyanex 302, as expected from the solvent extraction background. Maximum sorption capacities are affected by the extractant content in the capsules. The extractant fraction is generally lower in mononuclear-type capsules compared to matrix-type materials. As a consequence, maximum sorption capacities are generally higher in matrix-type sorbents. 

On the other hand the kinetics are controlled by the resistance to intraparticle diffusion: small particles reach equilibrium faster than large capsules. However, the modeling of kinetic profiles is well fitted by both the Crank equation (resistance to intraparticle diffusion) and the pseudo-second order rate equation. The intraparticle diffusion coefficient is about three orders of magnitude lower than the intraparticle diffusivity in water, as a confirmation of the resistance to intraparticle diffusion. The intraparticle diffusion coefficients are on the same order of magnitude for the mononuclear-type and matrix-type capsules.

Comparing the advantages and drawbacks of the different modes of encapsulation in terms of maximum sorption capacities (sorption isotherms) and mass transfer (uptake kinetics), the matrix-type capsules are found to be more appropriate.

Lead can be readily desorbed from metal-loaded Cyanex/alginate capsules (though with slow kinetics that require about 90 min for achieving about 95% of total desorption) using preferentially 1 M nitric acid solutions. However, a strong drawback appeared when recycling the sorbent: sorption efficiency for the next cycle strongly decreases, probably due to the oxidation of the extractant. The extent of the conversion was not analyzed; however, they are expected to produce Cyanex 302 (for Cyanex 301) and Cyanex 272 (for Cyanex 302) (based on the literature). The Cyanex 302 and Cyanex 272 extractants, being more sensitive to pH, require higher pH for metal cation extraction. The desorption efficiency and the recycling do not appear to be affected by the mode of encapsulation.

## Figures and Tables

**Table 1:** Characteristics of EIRs (Cyanex content and capsule size)—Effect of extractant loading, bead size, and mode of immobilization (N: mononuclear capsule; M: matrix immobilization).

Extractant	Mode	Extractant Concentration * (% v/v)	Size	Cyanex Content *q*_HA_ (mg·g^−1^)	Cyanex Content *q*_HA_ (µmol·g^−1^)	Capsule Size (µm)
C301	M	25	-	144.0 ± 2.5	446 ± 8	623 ± 14
C301	M	50	L	338.9 ± 10.8	1051 ± 33	1846 ± 39
C301	M	50	S	290.2 ± 4.7	900 ± 15	724 ± 19
C301	N	50	-	93.9 ± 4.5	291 ± 14	1110 ± 31
C302	M	50	-	314.5 ± 9.0	1026 ± 29	1430 ± 46
C302	M	75	-	480.9 ± 25.3	1569 ± 8	1378 ± 19
C302	N	50	-	55.8 ± 2.1	182 ± 7	876 ± 25
C302	N	75	-	203.8 ± 5.0	665 ± 16	1034 ± 54

* Cyanex concentration in organic phase (kerosene).

**Table 2:** Parameters of the Langmuir equation for Pb(II) sorption using Cyanex 301 and Cyanex 302 immobilized in alginate capsules.

Sorbent	Cyanex Content *q*_HA_ (µmol·g^−1^)	*q*_m_ (mg·Pb·g^−1^)	*q*_m_ (µmol·Pb·g^−1^)	*b* (L·mg^−1^)	*R*^2^	Cyanex/Pb(II) (mol/mol)
C301-M-25	446 ± 8	48.3	233	0.445	0.999	1.91
C301-M-50L	1051 ± 33	69.3	334	5.96	0.999	3.14
C301-M-50S	900 ± 15	78.6	379	3.44	0.999	2.37
C301-N-50	291 ± 14	36.3	175	1.06	0.998	1.66
C302-M-50	1026 ± 29	35.1	169	1.58	0.999	6.06
C302-M-75	1569 ± 8	49.5	239	1.83	0.997	6.57
C302-N-75	665 ± 16	24.2	117	0.47	0.998	5.69

**Table 3:** Parameters of the RIDE model (Crank equation) for Pb(II) sorption using Cyanex 301 and Cyanex 302 immobilized in alginate capsules.

Sorbent	Cyanex Content *q*_HA_ (µmol·g^−1^)	*D*_e_ × 10^11^ (m^2^·min^−1^)	MSR
C301-M-25	446 ± 8	7.95	0.077
C301-M-50L	1051 ± 33	1.60	0.036
C301-M-50S	900 ± 15	0.27	0.078
C301-N-50	291 ± 14	9.26	0.233
C302-M-50	1026 ± 29	2.40	0.049
C302-M-75	1569 ± 8	7.13	0.050
C302-N-75	665 ± 16	3.20	0.108

MSR: mean square of residuals.

**Table 4:** Parameters of the PFORE and PSORE models for Pb(II) sorption using Cyanex 301 and Cyanex 302 immobilized in alginate capsules.

Sorbent	*q*_HA_	*q*_e,exp_	PFORE			PSORE		
			*q*_e,1_	*k*_1_ × 10^2^	MSR	*q*_e,2_	*k*_2_ × 10^3^	MSR
C301-M-25	446	42.94	40.13	5.83	2.67	41.88	2.05	0.804
C301-M-50L	1051	64.76	61.60	0.42	25.0	65.03	0.10	8.016
C301-M-50S	900	76.88	73.06	2.26	17.0	76.99	0.45	1.333
C301-N-50	291	29.65	28.94	1.15	0.50	31.17	0.53	2.956
C302-M-50	1026	30.62	29.25	1.89	1.31	30.77	0.99	0.666
C302-M-75	1569	45.63	43.25	1.81	4.74	45.57	0.62	1.111
C302-N-75	665	24.69	22.46	3.10	1.06	23.60	1.96	0.831

Mean Cyanex content, *q*_HA_: (µmol g^−1^); *q*_e,exp_, *q*_e,1_, *q*_e,2_: mg·Pb·g^−1^; *k*_1_: min^−1^; *k*_2_: g·mg^−1^·min^−1^; MSR: mean square of residuals.

**Table 5:** Selection of the eluent for the desorption of Pb(II) from Cyanex 301 and Cyanex 302 immobilized in alginate capsules.

Sorbent	Desorption Efficiency (%)		
	0.1 M HNO_3_	1 M HNO_3_	1 M Thiourea/1 M HCl
C301-M-25	56.1	>99.9	88.2
C302-M-50	39.0	>99.9	83.1
C302-N-75	78.0	>99.9	85.9

Operating conditions—sorption and desorption: *SD*: 2 g·L^−1^; *t*: 24 h; *v*: 150 rpm; *T*: 20 °C/sorption: *C*_0_: 130 mg·Pb·L^−1^ except for C301-M-25 with *C*_0_: 200 mg·Pb·L^−1^.

**Table 6:** Efficiency of sorption and desorption (%) for three successive cycles for Pb(II) recovery using Cyanex 301 and Cyanex 302 immobilized in alginate capsules.

Sorbent	Cycle # 1		Cycle # 2		Cycle # 3	
	Sorption	Desorption	Sorption	Desorption	Sorption	Desorption
C301-M-25	35.5	97.7	2.9	94.6	2.1	93.5
C302-M-50	55.8	99.5	4.6	90.5	5.8	70.9
C302-N-75	33.2	98.6	4.9	6.6	-	-

Operating conditions—sorption and desorption: *SD*: 2 g·L^−1^; *t*: 24 h; *v*: 150 rpm; *T*: 20 °C/sorption: *C*_0_: 130 mg·Pb·L^−1^ except for C301-M-25 with *C*_0_: 200 mg·Pb·L^−1^; rinsing step between each cycle by contact with 0.01 M HCl solution for 5 min.

## Supplementary Materials

The following are available online at www.mdpi.com/1996-1944/10/6/634/s1: Figure S1: Chemical structure of Cyanex 301 and Cyanex 302.; Figure S2: Optical photographs of Cyanex 301 and Cyanex 302 extractants immobilized in alginate capsules; Figure S3: Example of SEM-EDX analysis of Cyanex 301 immobilized in alginate capsules (C301-N-50) (secondary electron image, inner and outer surfaces); Figure S4: Example of SEM-EDX analysis of Cyanex 302 immobilized in alginate capsules (C302-M-50) (secondary electron image, P element distribution map, distribution profile of P element across a section of the sorbent particle, and detail of internal porosity); Figure S5: Test of HDM and SCM with resistance to film diffusion, resistance to intraparticle diffusion and chemical reaction rate for Cyanex 301-M-25 sorbent; Figure S6: Test of HDM and SCM with resistance to film diffusion, resistance to intraparticle diffusion and chemical reaction rate for Cyanex 301-M-50L sorbent; Figure S7: Test of HDM and SCM with resistance to film diffusion, resistance to intraparticle diffusion and chemical reaction rate for Cyanex 301-M-50S sorbent; Figure S8: Test of HDM and SCM with resistance to film diffusion, resistance to intraparticle diffusion and chemical reaction rate for Cyanex 301-N-50 sorbent; Figure S9: Test of HDM and SCM with resistance to film diffusion, resistance to intraparticle diffusion and chemical reaction rate for Cyanex 302-M-50 sorbent; Figure S10: Test of HDM and SCM with resistance to film diffusion, resistance to intraparticle diffusion and chemical reaction rate for Cyanex 302-M-75 sorbent; Figure S11: Test of HDM and SCM with resistance to film diffusion, resistance to intraparticle diffusion and chemical reaction rate for Cyanex 302-N-75 sorbent; Table S1: Experimental conditions for the preparation of Cyanex/alginate capsules using the Büchi E-390 Encapsulator.
