# Supplementary material for: Extractant Immobilization in Alginate Capsules (Matrix- and Mononuclear-Type): Application to Pb(II) Sorption from HCl Solutions
Source: Materials (Basel). 2017 Jun 9;10(6):634. doi: 10.3390/ma10060634 (PMC5554015; doi:10.3390/ma10060634)
Supplement: Supplementary file 1 [file materials-10-00634-s001.pdf]

Supplementary Materials: Extractant immobilization in alginate capsules (matrix- and mononuclear-type): Application to Pb(II) sorption from HCl solutions

Janette Alba [1,2], Ricardo Navarro [1,*], Imelda Saucedo [1], Thierry Vincent [2] and Eric Guibal [2,*]

[1] Departamento de Química, División de Ciencias Naturales y Exactas, Universidad de Guanajuato, Guanajuato, CP 36040, Mexico; janetac8@hotmail.com (J.A.); sauceti@ugto.mx (I.S.)
[2] Centre des Matériaux des Mines d'Alès, Ecole des mines d'Alès, F-30319 Alès cedex, France; thierry.vincent@mines-ales.fr
* Correspondence: navarrm@ugto.mx (R.N.); eric.guibal@mines-ales.fr (E.G.); Tel.: +33-(0)466-782-734 (E.G.)

Table S1. Experimental conditions for the preparation of Cyanex/alginate capsules using the Büchi E-390 Encapsulator.

-	Cyanex 301				Cyanex 302			
Type of capsule*	N	M	M	M	N	N	M	M
Extractant concentration (in kerosene) (% v/v)	50	25	50L	50S	50	75	50	75
Alginate concentration (% w/w)	1.0	1.5	1.5	1.5	1.0	1.0	1.5	1.5
Alginate:organic phase (v/v)	-	13:7	13:7	13:7	-	-	13:7	13:7
Diameter of external nozzle (µm)	500	300	750	300	400	500	450	450
Diameter of internal nozzle (µm)	300	-	-	-	300	300	-	-
Vibration frequency (Hz)	200	300	200	200	200	200	200	200
Pressure (mbar)	88	256	305	315	117	193	250	285
Total flow rate (mL min^{-1})	15	10	16	6	13	12	20	24
Extractant flow rate (mL min^{-1})	1.8	-	-	-	2	3	-	-
Alginate flow rate (mL min^{-1})	13.2	-	-	-	11	9	-	-
External diameter of the beads (µm)	1110 ± 31	623 ± 14	1846 ± 39	724 ± 19	876 ± 25	1034 ± 54	1430 ± 46	1378 ± 19
Internal diameter of the beads (µm)	628 ± 6	-	-	-	391 ± 24	658 ± 3	-	-

* Type of capsule: mononuclear capsule (N) and matrix immobilization (M). [$CaCl_2$]: 0.5 M; T: 20 °C; amplitude: 3; electrostatic potential: 2500 V; agitation speed and time of agitation for preparation of emulsion in the synthesis of capsules with matrix immobilization: 11000 rpm and 10 min.

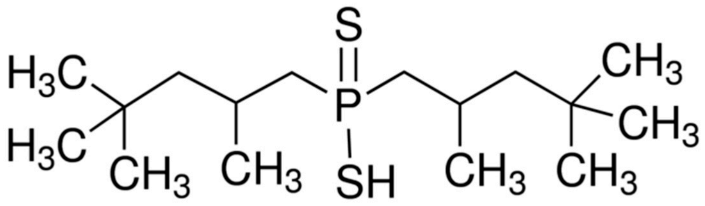

Cyanex 301

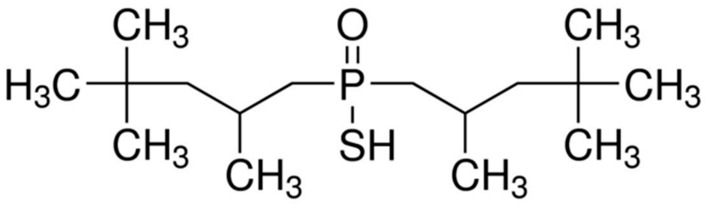

Cyanex 302

Figure S1. Chemical structure of Cyanex 301 and Cyanex 302.

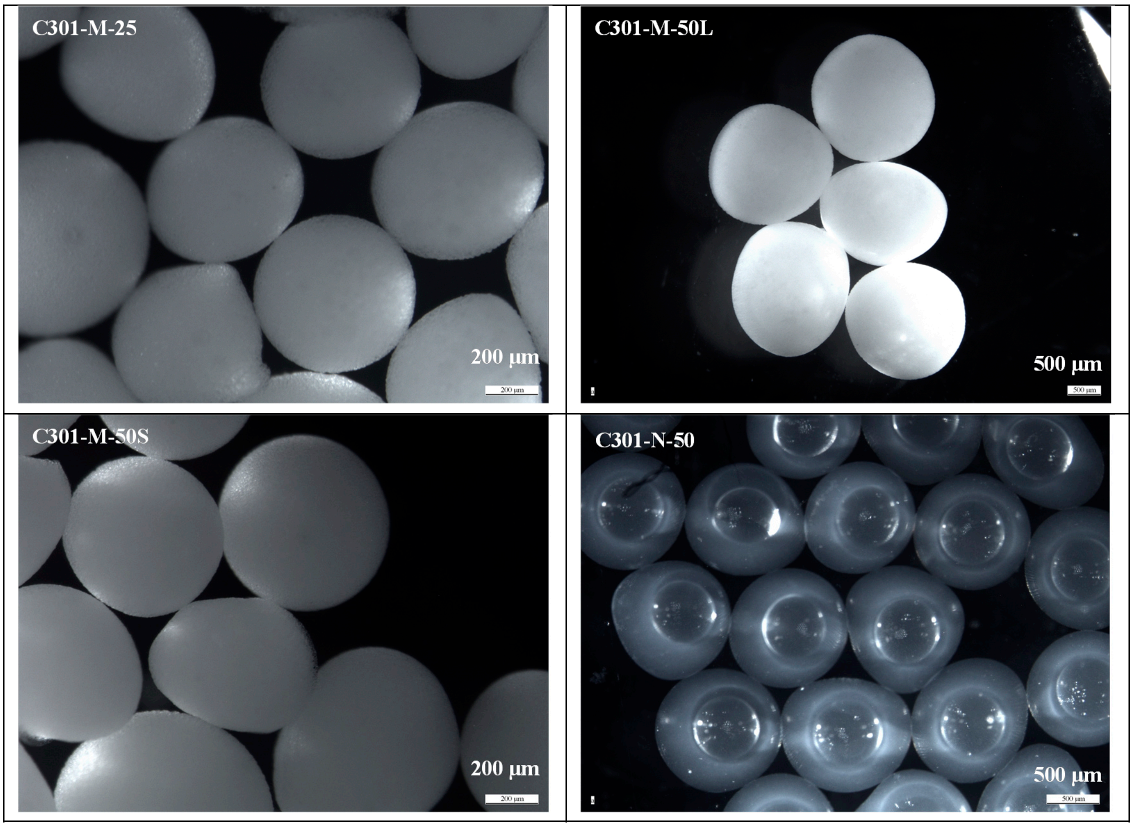

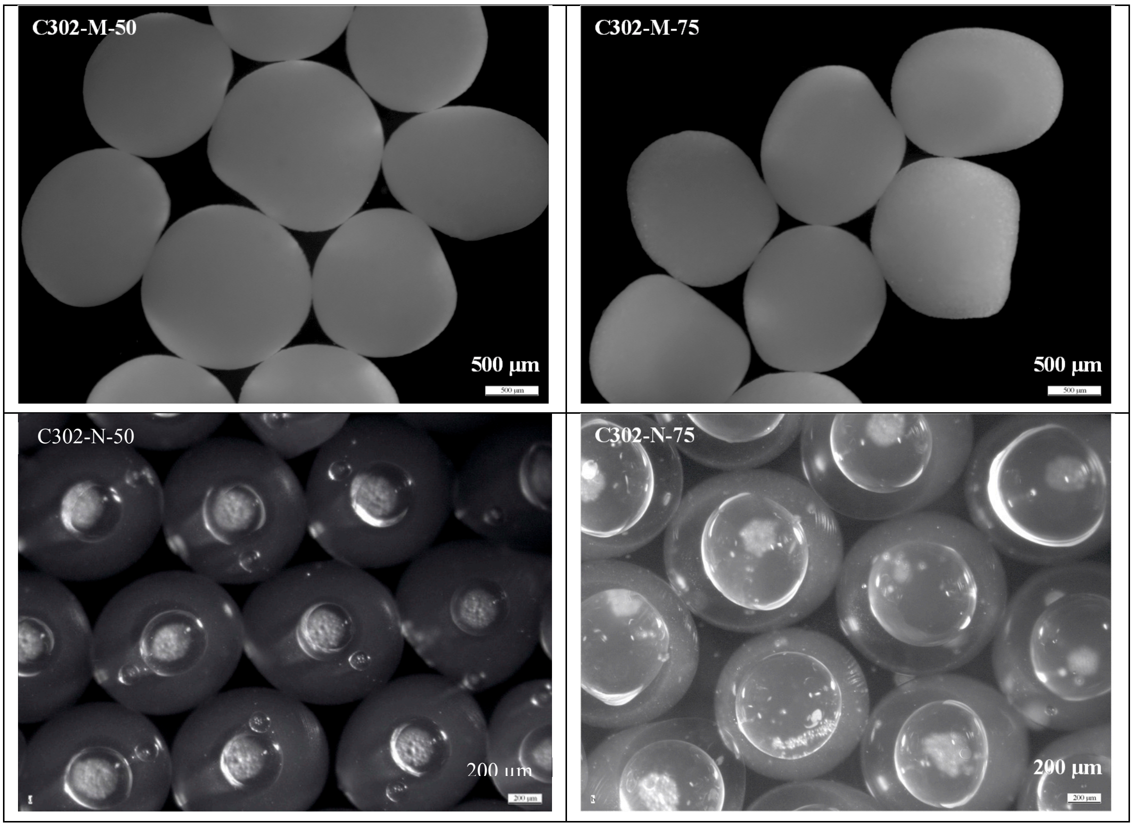

Figure S2. Optical photographs of Cyanex 301 and Cyanex 302 extractants immobilized in alginate capsules.

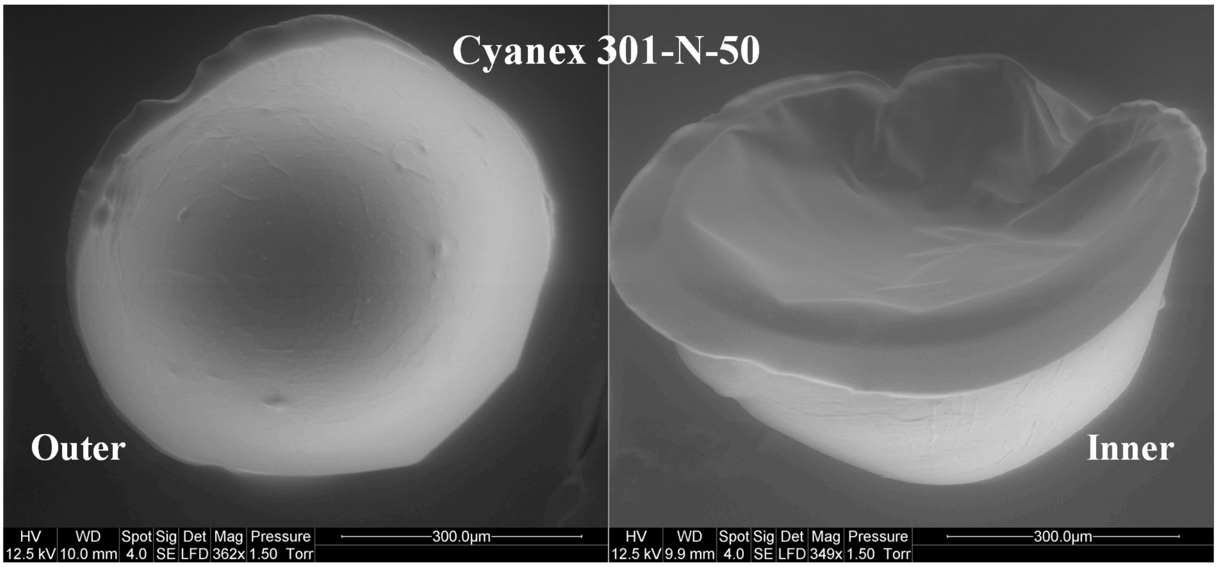

Figure S3. Example of SEM-EDX analysis of Cyanex 301 immobilized in alginate capsules (C301-N-50) (secondary electron image, inner and outer surfaces).

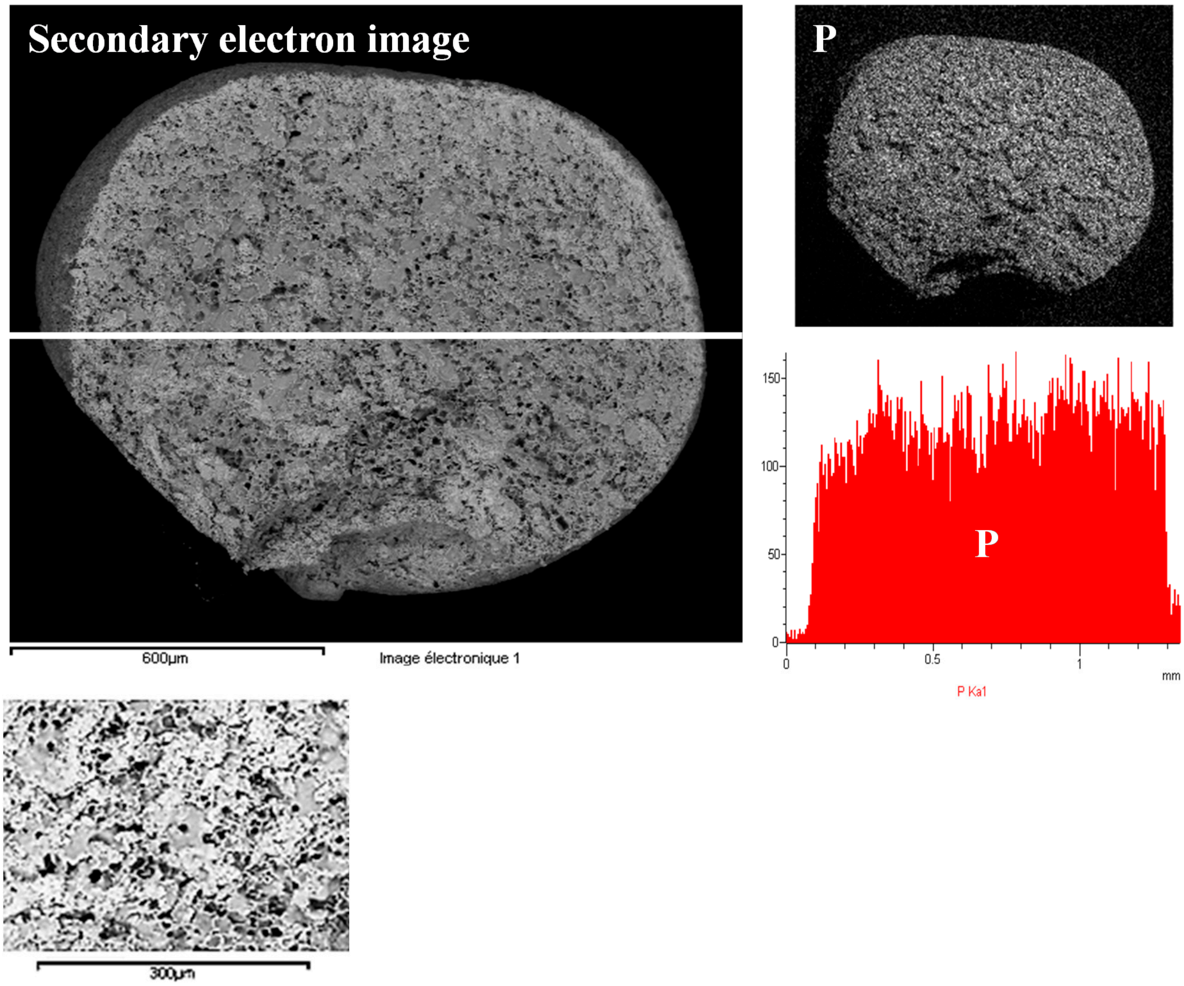

Figure S4. Example of SEM-EDX analysis of Cyanex 302 immobilized in alginate capsules (C302-M-50) (secondary electron image, P element distribution map, distribution profile of P element across a section of the sorbent particle, and detail of internal porosity).

Discussion of controlling steps in uptake kinetics

The uptake kinetics can be controlled by a series of mechanisms including the proper chemical reaction rate but also by diffusion mechanisms (including resistances to film diffusion and to intraparticle diffusion). The identification of the controlling step is important for optimizing the process; this allows selecting best experimental conditions or optimizing the design of the sorbent (for limiting, for example, resistance to intraparticle diffusion). Actually, the modeling of uptake kinetics should take into account all these steps, boundary conditions and constraints (film diffusion, intraparticle diffusion, reaction rate, equilibrium distribution …) at the expense of using complex numerical analysis systems [1]. Juang and Ju [2] discussed a series of simplified modeling systems derived from the homogeneous diffusion model (HDM) and the shrinking core model (SCM). The HDM involves counter-diffusion of exchangeable species in quasi homogeneous media, with a contribution from film diffusion (HDM-FD) and/or particle diffusion (HDM-PD). Solute molecules and exchangeable species (immobilized on the resin) follow a similar diffusion mechanism (but in the opposite direction). In the case of the SCM, a sharp virtual boundary exists between the reacted shell of the particle and the unreacted core, and this boundary moves towards the center of the particle [3,4]. This model was developed with different systems depending on the controlling step: film diffusion (SCM-FD), particle diffusion (SCM-PD) and chemical reaction rate (SCM-CR) [2]. A number of mathematical equations have been developed to simulate these mechanisms, they are listed below:

Homogeneous Diffusion Model

Film Diffusion:

$$F_1(X) = -\ln(1-X) = f(t), \qquad \text{(S1)}$$

Intraparticle Diffusion:

$$F_2(X) = -\ln(1-X^2) = f(t), \qquad \text{(S2)}$$

Shrinking Core Model

Film Diffusion:

$$G_1(X) = X = g\left(\int_0^t C(t)dt\right), \qquad \text{(S3)}$$

Intraparticle Diffusion:

$$G_2(X) = 3 - 3(1-X)^{2/3} - 2X = g\left(\int_0^t C(t)dt\right), \qquad \text{(S4)}$$

Chemical Reaction Rate:

$$G_3(X) = 1 - (1-X)^{1/3} = g\left(\int_0^t C(t)dt\right), \qquad \text{(S5)}$$

Where X is the fractional approach to equilibrium (i.e., $q(\mathrm{t})/q_{\mathrm{eq}}$), the amount adsorbed at time t divided by the amount of metal adsorbed at equilibrium. Plotting Fi and Gi functions versus time and the integral term (respectively) determined the most appropriate mechanism for describing the controlling step. The curve giving a straight line (good correlation measured by the correlation coefficient) is the predominant limiting step.

Equations (S1–S5) have been tested for each experimental series (Figures S5–S11). In most cases several sections of linear distribution were observed: an initial section (within the first 2–5 hours) corresponding to a fast sorption of Pb(II), followed by a long and slow sorption phase that may take place for several days. This clearly means that several mechanisms were involved in the control of uptake mechanisms including different diffusion regimes and/or variable intraparticle diffusion control. The first phase counts for about 80%–90% of total sorption while the slow phase corresponds to the resistance to intraparticle diffusion in poorly diffusive compartment of the composite material. Left panels in Figures S5–S11 show the representation of mathematical functions $F\mathrm{i}(X)$ and $G\mathrm{i}(Y)$ for complete uptake kinetics while right panels represent the functions in the initial section of uptake kinetics (i.e., 90–240 min). The analysis of the initial sections with the different equations shows very different behaviors for the different systems. In most cases the best fit of experimental data was obtained with the mechanisms involving a resistance to intraparticle diffusion and in general the correlation coefficient was slightly higher for the SCM equation than for HDM equation. Frequently the models fail to describe the very first minutes of contact (probably due to a more marked contribution of resistance to film diffusion). However, some specific cases can be identified. Hence for Cyanex 301-M-25, it was not possible identifying a model that linearly fits experimental data. On the opposite hand, for Cyanex 301-N-50 and Cyanex 302-M-50 all the equations show very similar fitting (though the correlations coefficients were relatively low); this means that complex combined mechanisms may take place in the control of uptake kinetics.

These tests mean that the resistance to intraparticle diffusion represents the most significant limiting step in the mass transfer, though the contribution of other parallel mechanisms are also taking place (film diffusion, and chemical reaction rate). The discrepancies observed for some of these EIRs cannot be directly associated to a given extractant or a preferred synthesis mode (M vs. N).

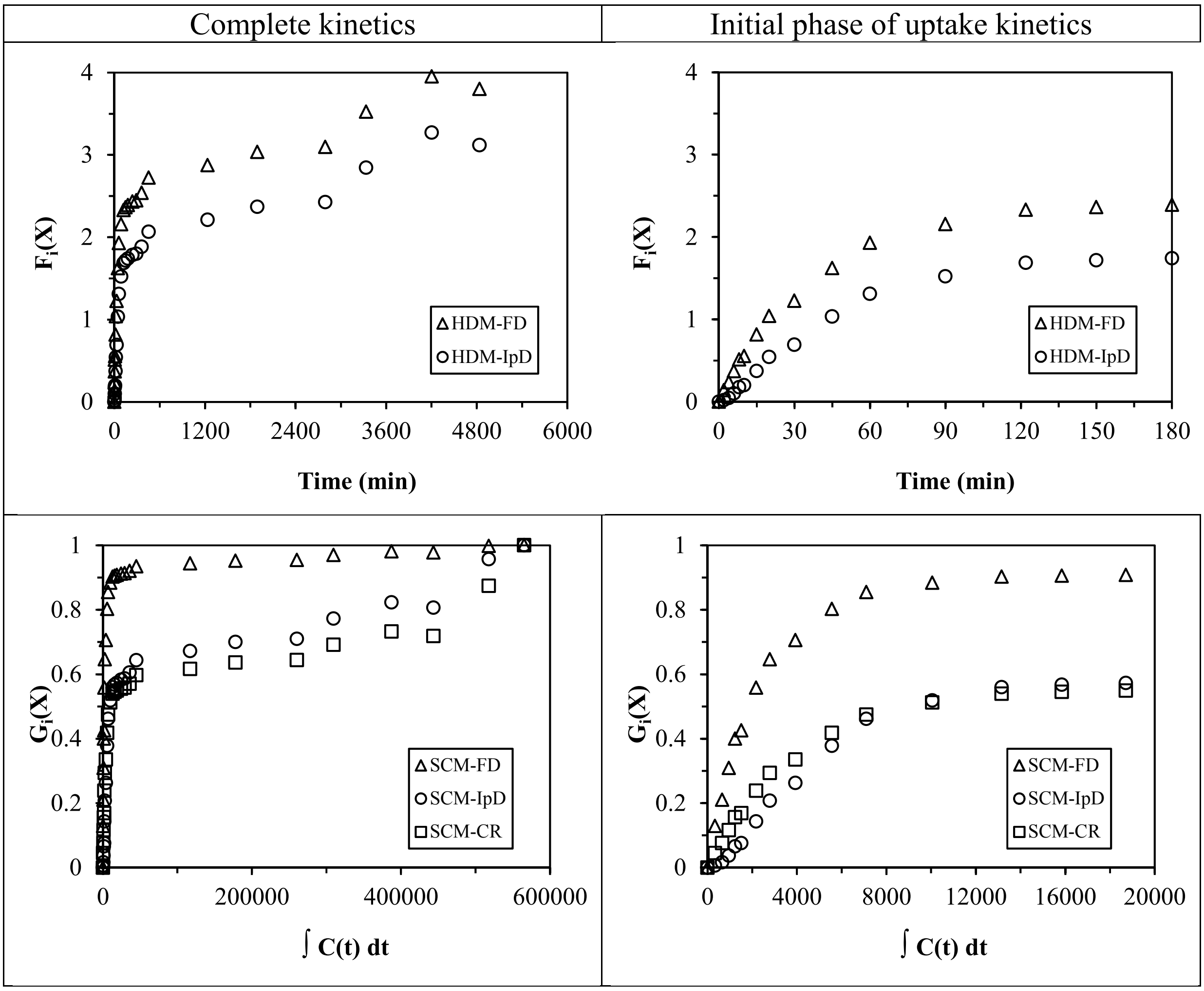

Figure S5. Test of HDM and SCM with resistance to film diffusion, resistance to intraparticle diffusion and chemical reaction rate for Cyanex 301-M-25 sorbent.

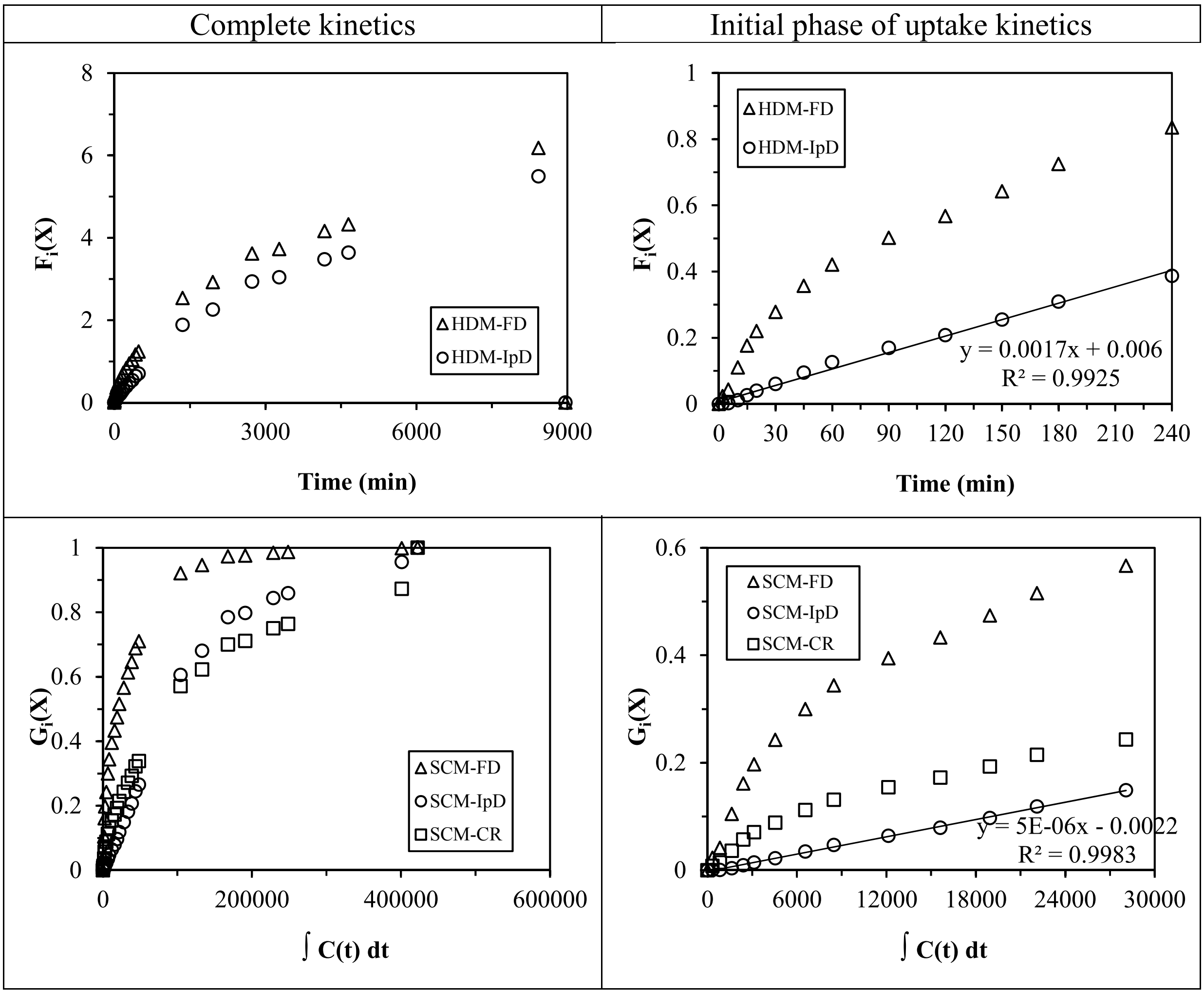

Figure S6. Test of HDM and SCM with resistance to film diffusion, resistance to intraparticle diffusion and chemical reaction rate for Cyanex 301-M-50L sorbent.

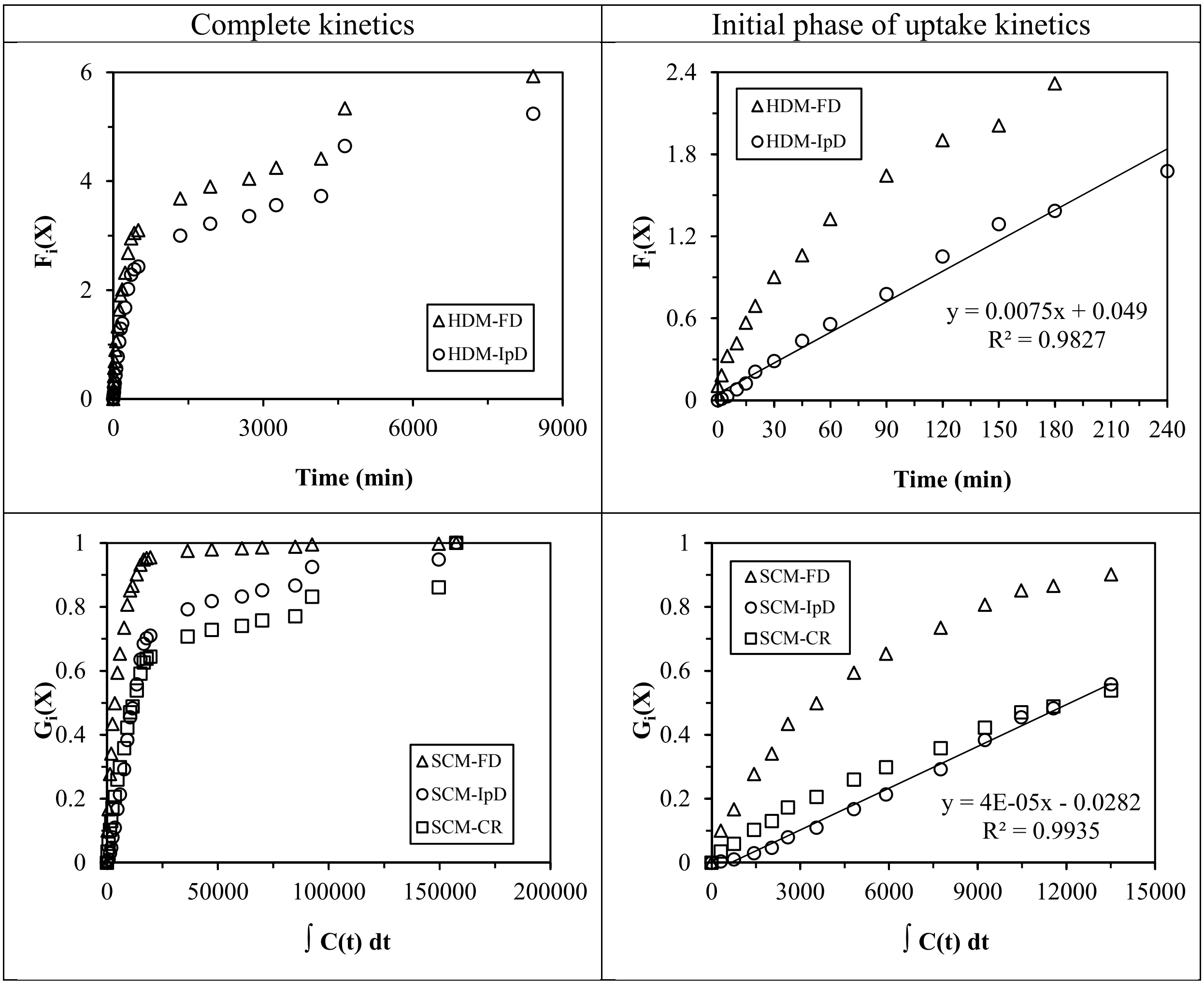

Figure S7. Test of HDM and SCM with resistance to film diffusion, resistance to intraparticle diffusion and chemical reaction rate for Cyanex 301-M-50S sorbent.

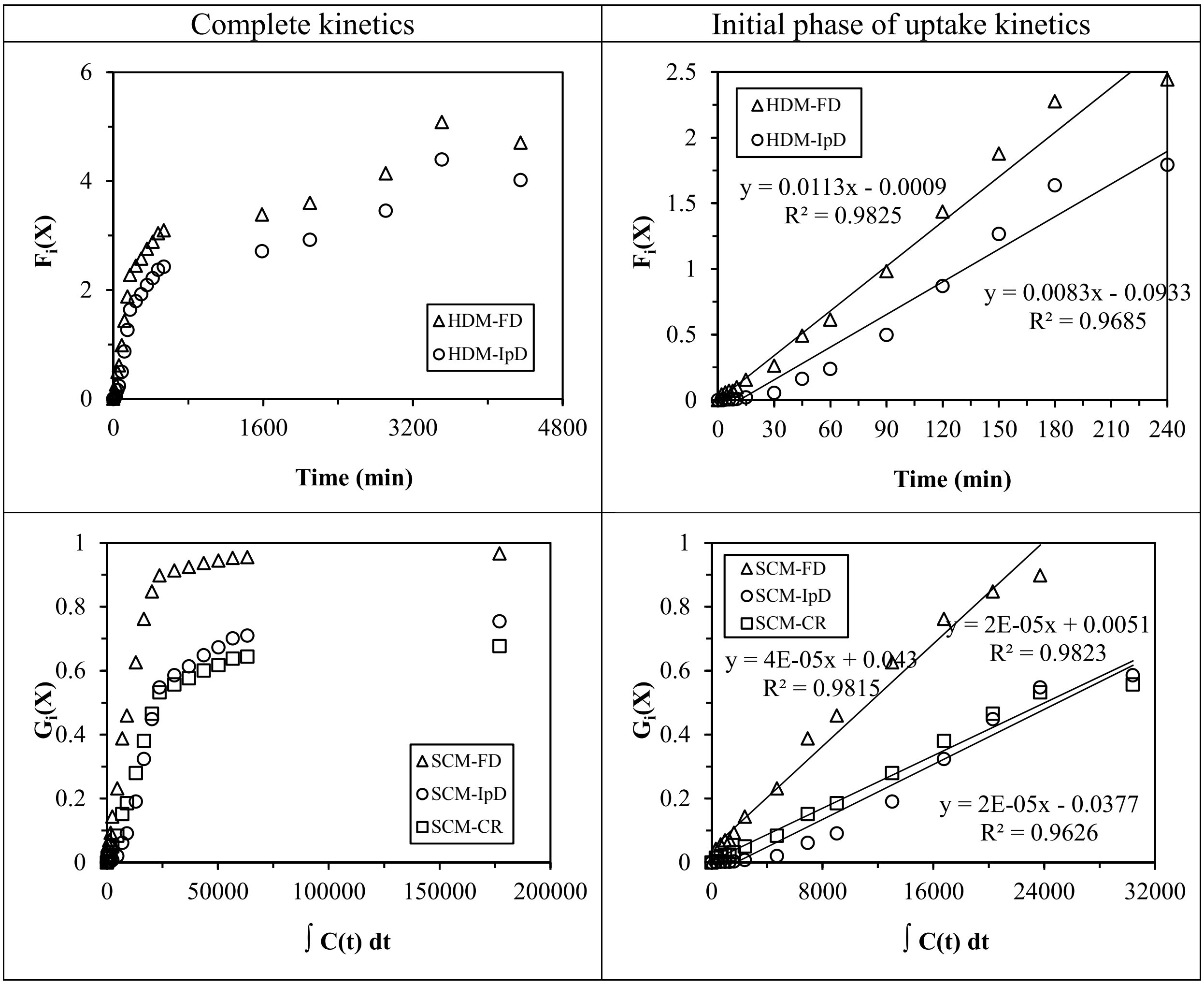

Figure S8. Test of HDM and SCM with resistance to film diffusion, resistance to intraparticle diffusion and chemical reaction rate for Cyanex 301-N-50 sorbent.

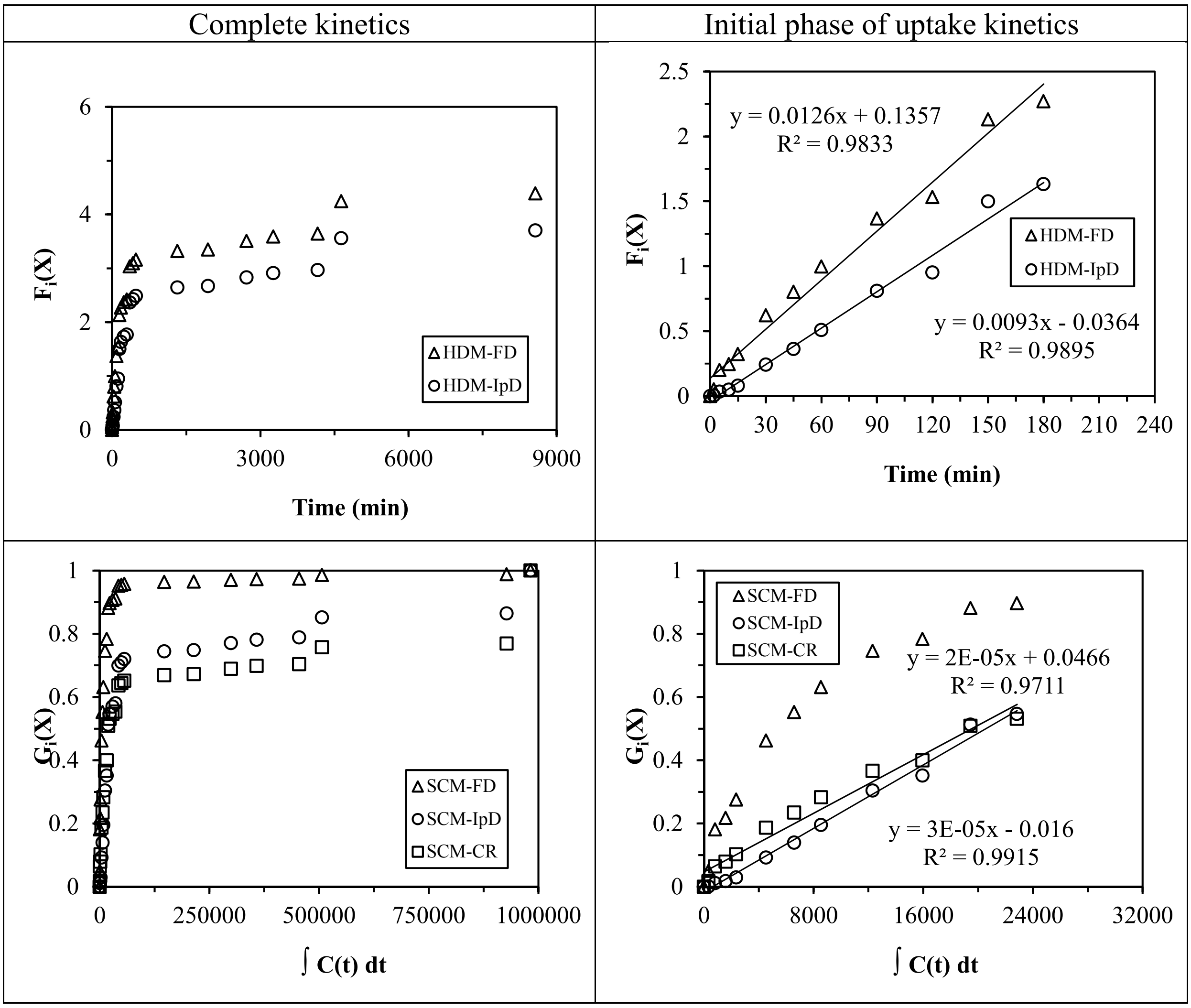

Figure S9. Test of HDM and SCM with resistance to film diffusion, resistance to intraparticle diffusion and chemical reaction rate for Cyanex 302-M-50 sorbent.

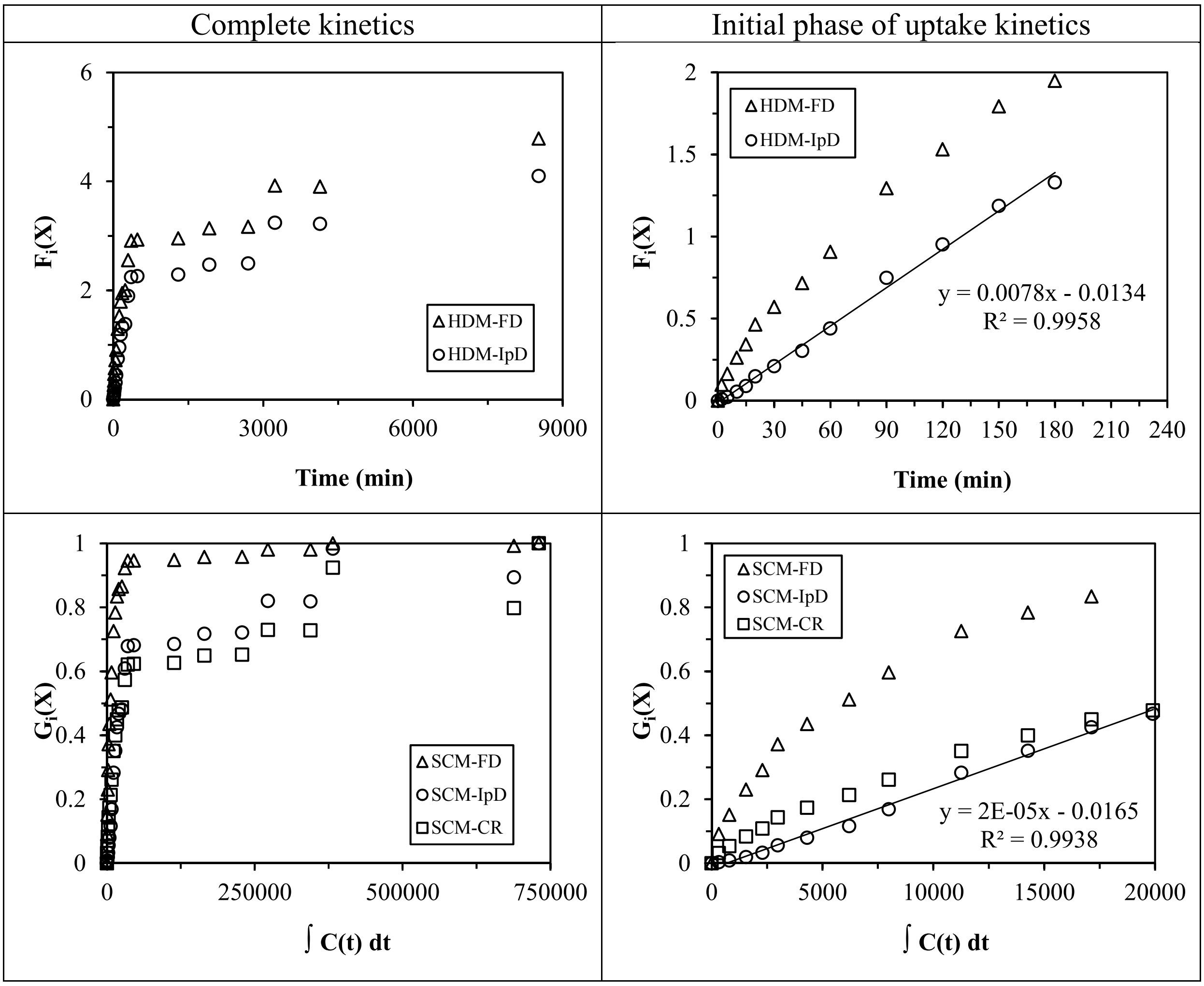

Figure S10. Test of HDM and SCM with resistance to film diffusion, resistance to intraparticle diffusion and chemical reaction rate for Cyanex 302-M-75 sorbent.

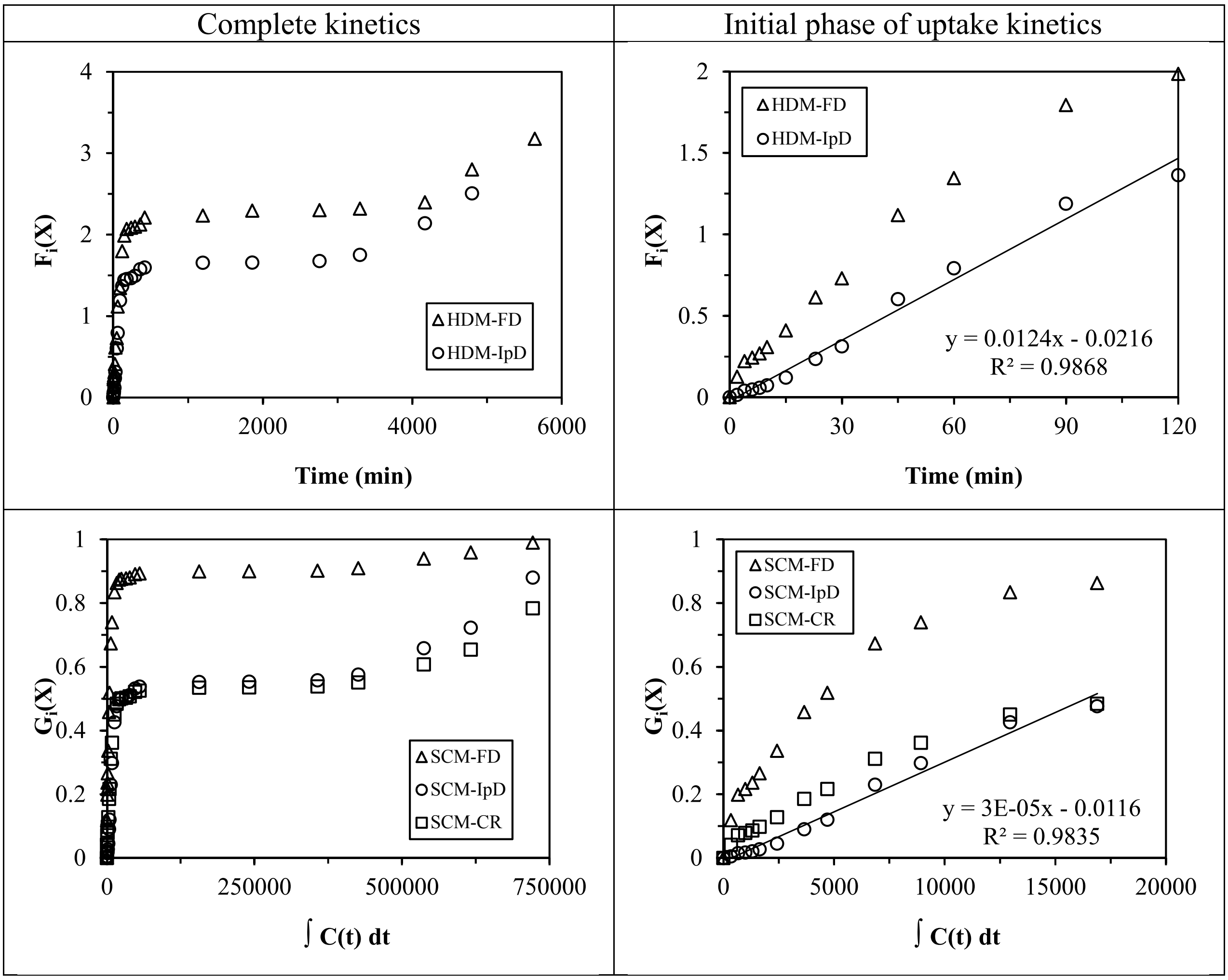

Figure S11. Test of HDM and SCM with resistance to film diffusion, resistance to intraparticle diffusion and chemical reaction rate for Cyanex 302-N-75 sorbent.

References

1. Tien, C. *Adsorption Calculations and Modeling*; Butterworth-Heinemann: Newton, MA, USA, 1994.
2. Juang, R.-S.; Ju, C.-Y. Kinetics of sorption of Cu(II)-ethylenediaminetetraacetic acid chelated anions onto cross-linked, polyaminated chitosan beads. *Ind. Eng. Chem. Res.* **1998**, *37*, 3463–3469.
3. Juang, R.-S.; Lin, H.-C. Metal sorption with extractant-impregnated macroporous resins. 1. Particle diffusion kinetics *J. Chem. Technol. Biotechnol.* **1995**, *62*, 132–140.
4. Juang, R.-S.; Lin, H.-C. Metal sorption with extractant-impregnated macroporous resins. 2. Chemical reaction and particle diffusion kinetics *J. Chem. Tech. Biotechnol.* **1995**, *62*, 141–147.
